# Spontaneous subepithelial hemorrhage: the Antopol-Goldman lesion

**DOI:** 10.31744/einstein_journal/2021AI5829

**Published:** 2021-01-27

**Authors:** Eduardo Kaiser Ururahy Nunes Fonseca, Roberto Vitor Almeida Torres, Luiz Raphael Pereira Donoso Scoppetta, Cesar Higa Nomura

**Affiliations:** 1 Hospital das Clínicas Faculdade de Medicina Universidade de São Paulo São PauloSP Brazil Instituto do Coração, Hospital das Clínicas, Faculdade de Medicina, Universidade de São Paulo, São Paulo, SP, Brazil.

A 55-year-old man admitted to our emergency department complaining of right flank acute pain. He also referred epistaxis, gingival bleeding, and hematuria for the past 3 days. His physical exam demonstrated abdominal subcutaneous hematomas and he referred abdominal tenderness on palpation. Seven days before these symptoms, his daily warfarin dose has been increased. His blood tests showed that the International Normalized Ratio was markedly elevated (3.6; his previous exam from before dosage increase was 2.2).

**Figure 1:**
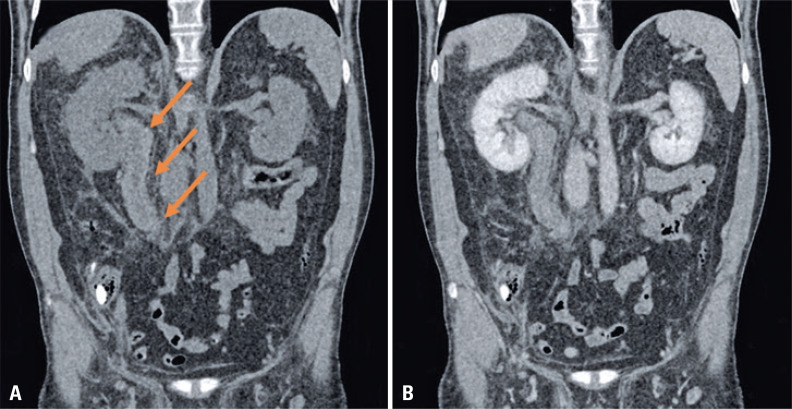
Abdominal computed tomography in coronal view. (A) unenhanced image shows a hyperattenuating circumferential thickening of the right ureter wall in practically its entire extension with adjacent fat-stranding; (B) contrast-enhanced image shows no enhancement of the ureteral wall. These findings are compatible with an intramural hematoma. It should be highlighted that given the hyperattenuation of the subepithelial hematoma, it should be mistaken by a diffuse enhancement of the ureteral wall if the non-contrast phase is not performed

An abdominal computed tomography was requested (Figure 1) and showed hyperattenuating and non-enhancing circumferential thickening of the right ureter wall, compatible with extensive parietal hematoma.

Spontaneous subepithelial hemorrhage is a rare cause of abdominal pain and hematuria ^(1-3)^ and this is usually associated with bleeding diathesis, mostly from anticoagulant therapy, as seen in our case. This bleeding is also called “Antopol-Goldman lesion” after the first description of this entity. Unenhanced abdominal computed tomography is of great help for this difficult diagnosis as it reveals a spontaneously hyperattenuating^(1,4-6)^ thickening of the renal pelvis and/or ureteral wall. This finding may be difficult to differentiate from contrast enhancement in contrast-enhanced images, making unenhanced phases a must for this diagnosis.

Both urologists and radiologists should be aware of this benign as it may mimic urothelial neoplasms and lead to unnecessary nephrectomy, approximately 30% in the first description.^(3)^ Emergency radiologists must maintain a high grade of suspicion, primarily in patients using anti-coagulants as this diagnosis is rarely suspected before image.^(1-6)^ Spontaneous subepithelial hemorrhage normally resolve spontaneously after anti-coagulants discontinuation and its association with hydronephrosis or urinary obstruction is rare.^(1)^
