# Supplementary material for: Spontaneous subepithelial hemorrhage: the Antopol-Goldman lesion
Source: Einstein (Sao Paulo). 2021 Jan 27;19:eAI5829. doi: 10.31744/einstein_journal/2021AI5829 (PMC7839946; doi:10.31744/einstein_journal/2021AI5829)
Supplement: Translation

# Translation: Hemorragia subepitelial espontânea: lesão de Antopol-Goldman

**Authors:** Eduardo Kaiser Ururahy Nunes Fonseca, Roberto Vitor Almeida Torres, Luiz Raphael Pereira Donoso Scoppetta, Cesar Higa Nomura

**Affiliations:** 1 Brasil Instituto do Coração, Hospital das Clínicas, Faculdade de Medicina, Universidade de São Paulo, São Paulo, SP, Brasil.

Homem, 55 anos, admitido em serviço de emergência relatando dor aguda no flanco direito. O paciente também se queixava de epitaxe, sangramento gengival e hematúria ao longo dos últimos 3 dias. O exame físico demonstrou hematoma subcutâneo no abdome, com dor à palpação. Sete dias antes desses sintomas, sua dose diária de varfarina fora aumentada. O exame sanguíneo mostrou que a Razão Normalizada Internacional estava marcadamente elevada (3,6; o exame prévio, anterior ao aumento da dosagem, mostrou o índice de 2,2).

Foi solicitada tomografia computadorizada abdominal (Figura 1), que expôs hiperatenuação e espessamento circunferencial da parede do ureter direito na fase sem contraste, compatível com hematoma expansivo parietal.

**Figura 1:** Tomografia computadorizada abdominal no plano coronal. (A) imagem sem contraste mostra espessamento parietal circunferencial hiperatenuante em praticamente toda extensão do ureter direito com densificação da gordura adjacente; (B) imagem com contraste não demonstra realce adicional na parede do ureter. Esses achados são compatíveis com hematoma intramural. Deve-se enfatizar que, devido à hiperatenuação do hematoma subepitelial, tal achado pode ser confundido com realce difuso da parede ureteral quando a fase sem contraste não é realizada

A hemorragia subepitelial espontânea é uma causa rara de dor abdominal e hematúria^(1-3)^ e, normalmente, está associada à diátese hemorrágica, principalmente por terapia anticoagulante, como observado em nosso caso. Esse sangramento também é chamado de “lesão de Antopol-Goldman” após a primeira descrição dessa entidade. A tomografia computadorizada abdominal sem contraste é de grande ajuda para esse diagnóstico, já que demonstra a hiperatenuação espontânea^(1,4-6)^ da pelve renal e/ou parede ureteral. Esse achado pode dificultar o diferencial por imagem, caso só seja realizada a fase com contraste, de modo que a fase sem contraste é imprescindível para o diagnóstico.

Tanto os urologistas quanto os radiologistas devem reconhecer esse achado benigno, já que ele pode simular uma neoplasia urotelial, levando à nefrectomia desnecessária em até 30% dos casos, segundo sua primeira descrição.^(3)^ Os radiologistas de emergência devem manter um alto grau de suspeita, principalmente em pacientes utilizando anticoagulantes, considerando que esse diagnóstico é raramente lembrado antes de exame de imagem.^(1-6)^ Em geral, a hemorragia subepitelial espontânea é resolvida de forma natural após a interrupção do uso de anticoagulantes, e sua associação à hidronefrose e a obstrução urinária são raras.^(1)^
